# Nanostructured Lipid Carriers (NLC) for Biologically Active Green Tea and Fennel Natural Oils Delivery: Larvicidal and Adulticidal Activities against *Culex pipiens*

**DOI:** 10.3390/molecules27061939

**Published:** 2022-03-17

**Authors:** Ibrahim Taha Radwan, Mohamed M. Baz, Hanem Khater, Abdelfattah M. Selim

**Affiliations:** 1 Supplementary General Sciences Department, Faculty of Oral and Dental Medicine, Future University in Egypt, Cairo 11835, Egypt; ibrahim80radwan@hotmail.com; 2 Department of Entomology, Faculty of Science, Benha University, Benha 13518, Egypt; mohamed.albaz@fsc.bu.edu.eg; 3 Department of Parasitology, Faculty of Veterinary Medicine, Benha University, Toukh 13736, Egypt; hanemkhater2021@gmail.com; 4 Department of Animal Medicine (Infectious Diseases), College of Veterinary Medicine, Benha University, Toukh 13736, Egypt

**Keywords:** *Culex pipiens*, green tea oil, fennel oil, nanostructured lipid carriers nanoformulations

## Abstract

(1) Background: The control of mosquitoes with essential oils is a growing demand. (2) Methods: This study evaluated the novel larvicidal and adulticidal activity of fennel and green tea oils and their nanostructured lipid carriers (NLC) against *Culex pipiens* (*C. pipiens*) in the laboratory, field conditions and evaluated their effect against non-target organisms. SLN type II nanoformulations were synthesized and characterized using dynamic light scattering (DLS), zeta potential and transmission electron microscope. (3) Results: The synthesized NLCs showed spherical shaped, homogenous, narrow, and monomodal particle size distribution. The mortality percent (MO%) post-treatment (PT) with 2000 ppm for 24 h with fennel oil and NLC fennel (NLC-F) reached 85% (LC_50_ = 643.81 ppm) and 100% (LC_50_ = 251.71), whereas MO% for green tea oil and NLC green tea (NLC-GT) were 80% (LC_50_ = 746.52 ppm) and 100% (LC_50_ = 278.63 ppm), respectively. Field trial data showed that the larval reduction percent of fennel oil and NLC-F reached 89.8% and 97.4%, 24 h PT and the reduction percent of green tea oil and NLC-GT reached 89% and 93%, 24 h PT with persistence reached 8 and 7 days, for NLC-F and NLC-GT, respectively. The adulticidal effects showed that NLC-F and NLC-GT (100% mortality) were more effective than fennel and green tea oils (90.0% and 83.33%), with 24 h PT, respectively. Moreover, their reduction of adult density after spraying with LC_95_ X2 for 15 min, with fennel oil, NLC-F, and green tea oil, NLC-GT were 83.6%, 100%, 79.1%, and 100%, respectively, with persistence (>50%) lasting for three days. The predation rate of the mosquitofish, *Gambusia affinis*, and the bug, *Sphaerodema urinator*, was not affected in both oil and its NLC, while the predation rate of the beetle, *Cybister tripunctatus* increased (66% and 68.3%) by green tea oil and NLC-GT, respectively. (4) Conclusions: NLCs nanoformulation encapsulated essential oils was prepared successfully with unique properties of size, morphology, and stability. In vitro larvicidal and adulticidal effects against *C. pipiens* supported with field evaluations have been performed using essential oils and their nanoformulations. The biological evaluation of nanoformulations manifested potential results toward both larvicidal and adulticidal compared to the essential oils themselves, especially NLC encapsulated fennel oil which had promising larvicidal and adulticidal activity.

## 1. Introduction

One of the main goals of drug nanodelivery is to design an effective nanodelivery system for therapeutically active molecules, in order to minimize the disintegration by early degradation of the drug and maintain the optimal dose of the drug in the target tissue. The goal is to obtain the desired healing with minimal or no undesirable side effects [1]. Due to several limitations of drug administration, such as increased chances of missing doses, low bioavailability, hesitancy in drug level, rapid drug metabolism and toxicity, prolonged treatment time and its impact on poor patient pliability, and undesirable side effects, there has been a rise in anticipating nanotechnology and drug delivery rather than the traditional methods. Most of these problem are related to the hydrophilic and hydrophobic nature of the drugs that lead to the idea of drug encapsulation or drug delivery using nanocarriers like lipid nanoparticles and polymeric nanoparticles [2]. Solid Lipid Nanoparticles (SLN) and Nanostructured Lipid Carriers (NLC) are two different generations of SLN, they have comparable chemical constituents; the main difference is, the lipid matrix is composed not only of a solid lipid, but of a blend of a solid and a liquid lipid oil LNC formulation to enhance active ingredient solubility and enhance loading capacity. Other effective vesicles are used like, liposomes [3], ethosomes [4], niosomes [5], colloidosomes [6], herbosomes [7], bilosomes [8], pharmacosomes [9], sphingosomes [10] and transferosomes [11]. Many vesicles were formulated, especially after discovering nonionic-surfactant, a variety of vesicles formed [12]. Lipid-based nanocarriers have different drug delivery and physicochemical features. After drug delivery, hydrophobic drugs acquired good solubility and bioavailability, diminished dose by sustained and controlled release of drugs, and low drug toxicity. Furthermore, physicochemical features like small particle sizes varying from 50 nm to micrometer enhanced the ability to cross different biological boundaries [13,14].

Mosquitoes are dangerous pests to human and animal health because they are vectors for diseases like malaria, filarial nematodes, encephalitis, and the West Nile virus [15,16,17,18,19]. Consequently, mosquito control is a critical requirement worldwide, mainly where freshwater sources predominate [20]. In Egypt, the mosquito *Culex pipiens* (*C. pipiens*) (Diptera: Culicidae) is the main vector of filariasis in humans as well as many other animal diseases [21,22,23].

Synthetic pesticides are commonly used for mosquito control as a result of mosquito-spread and the concern of transmitting diseases. However, their widespread use has led to pest resistance, pollution, and health problems for humans and non-target organisms. So, the natural alternatives to synthetic pesticides have attracted medical and environmental attention around the world due to their biodegradability, low toxicity, and ability to overcome insecticide resistance [24,25].

Insecticides based on botanical extracts and essential oils are growing in popularity among organic producers and environmentally conscious consumers due to their acceptance in urban environments, homes, and other sensitive locations [26]. Furthermore, essential oils (EOs) are excellent in controlling various insect pests and are the most widely used locally and globally [27]. Tea leaves, *Camellia sinensis* are used to make a non-alcoholic beverage that is popular all over the world because of its psychotropic and health effects [28], whereas the young tea leaves contain many active secondary compounds such as caffeine, theobromine, theophylline, catechins, flavonoids, and glycosides and are widely evaluated as larvicidal against wide of insects. The major components of EOs include monoterpenes, alkaloids, physiologically related phenols, and sesquiterpenes [29,30]. This study aimed to evaluate the larvicidal and adulticidal effect of fennel and green tea oils and their novel NLC formulation against *C. pipiens* in vitro and field evaluations besides testing their efficacy against non-target predators for the first time.

## 2. Materials and Methods

### 2.1. Chemical and Oils

Stearic acid, Tween 20, sodium glycolate, sodium taurocholate, butanol, and decarbonated water were purchased from Alfa Aesar, Germany. Two essential oils, *Foeniculum vulgare* (fennel oils) and *Camellia sinensis* (green tea) were purchased from EL CAPTAIN Company for extracting natural oils, plants, and cosmetics (Cap Pharm, Cairo, Egypt). All chemicals were used without further purification.

### 2.2. Synthesis of Free (Unloaded) Lipid Nanostructured Carriers (SLN Type 2)

The lipid nanostructured carriers or SLN type 2 was synthesized according to the method described in the USOOS188837 patent by [31] with minor modifications as follows: about 2 g of stearic acid were heated in 50 mL beaker to its melting point 68.8 °C (up to 70 °C) until clear molten was obtained and kept heated to this temperature (solution I). Five mL distilled water, 2.5 mL Tween 20 and 0.2 mL butanol was added to a well-stirred mixture of 0.7 g sodium glycolate and 0.7 g sodium taurocholate dissolved in 10 mL water and the total volume became 15 mL, then heated to 70 °C (solution II).

The (solution I) was poured into (solution II) at the same temperature with stirring until a clear microemulsion solution was obtained. The microemulsion was rapidly quenched with 150 mL ice-cold water and dispersed using a probe sonicator for 20 min (400 W, time interval 4 s), producing lipid microsphere dispersion. Mannitol or sucrose was added to the dispersion as a cryoprotectant to obtain a semi-solid substance through lyophilization for two days at −45 °C.

### 2.3. Synthesis of Oil-Loaded Nanostructured Lipid Carriers

#### 2.3.1. Synthesis of Fennel Oil-Loaded Nanostructured Lipid Carriers

The oil-loaded NLC was synthesized according to the same method mentioned above as follows, in 50 mL beaker about 2 g of stearic acid and 2 g of fennel oil (immiscible in water) was added and heated up to 70 °C until clear molten obtained and kept heated to this temperature using a digital thermometer (solution I). In another 50 mL beaker, 5 mL distilled water, 2.5 mL Tween 20 and 0.2 mL butanol was added to a well-stirred and heated mixture of 0.7 g sodium glycolate and 0.7 g sodium taurocholate dissolved in 10 mL water then heated to 70 °C (solution II).

The (solution I) was poured into (solution II) at the same temperature with stirring until a clear microemulsion solution was obtained, which in turn quenched and dispersed using ultrasonic probe sonicator for 20 min in an ice-cooled water bath (400 W, time interval 4 s) producing lipid microsphere dispersion. Mannitol or sucrose was added to the dispersion as a cryoprotectant to obtain a semi-solid substance through lyophilization for two days at −45 °C.

#### 2.3.2. Synthesis of Green Tea Oil-Loaded Nanostructured Lipid Carriers

The green tea oil-loaded NLC was synthesized according to the same method mentioned above but the difference is that green tea oil is completely miscible in water.

Two g of stearic acid was heated in a 50 mL beaker above its melting point (up to 70 °C) until clear molten was obtained and kept heated to this temperature (solution I). Five mL distilled water, 2.5 mL Tween 20, 0.2 mL butanol and 2 g of green tea oil, was added to a well-stirred mixture of 0.7 g sodium glycolate and 0.7 g sodium taurocholate dissolved in 10 mL water then heated to 70 °C (solution II).

The (solution I) was poured into (solution II) at the same temperature with stirring until a clear microemulsion solution was obtained. The microemulsion was rapidly quenched with 150 mL ice-cold water and dispersed using a probe sonicator for 20 min (400 W, time interval 4 s), producing lipid microsphere dispersion. Mannitol or sucrose was added to the dispersion as a cryoprotectant to obtain a semi-solid substance through lyophilization for two days at −45 °C.

### 2.4. Characterization of Free NLC and NLC-Loaded Essential Oils

#### 2.4.1. Droplet Size and Surface Charge

The hydrodynamic radius and polydispersity index (PDI) were investigated by dynamic light scattering (DLS) at an angle of 173° in room temperature. The surface charge or zeta potential was measured by the frequency shift of scattered light at a scattering angle of 12°. Radius and PDI measured with (Zetasizer Nano ZS, Malvern Instruments Ltd., Malvern, UK) in the Egyptian petroleum research institute (EPRI). While, Zeta potential was measured by (NanoBrook Zetapals particle sizing software ver.5.23, Brookhavens Instrument corp., Holtsville, NY, USA) About 5 mg of each powder was dispersed in 10 mL of distilled water at a temperature of 25 °C.

#### 2.4.2. NLC Surface Morphology by Transmission Electron Microscope (TEM)

The morphology and structure visualization of NLCs was performed by field transmission electron microscopy (HR-TEM, JSM-7100F) in the Egyptian petroleum research institute (EPRI), cairo; images were recorded with JEOL JEM-2100-115 high-resolution transmission electron microscopes with accelerating voltage 200 kV. Nearly, 1 µL of NLCs was diluted with double distilled (1:200) and placed on a 300 mesh carbon-coated grid and attain for 2 min. the excess liquid was adsorbed by a cellulose filter. A drop of 2% (*w*/*w*) phosphotungstic acid (PTA) was applied to the grid for 10 s. to achieve negative staining the excess PTA was disposed of via adsorption on filter paper.

### 2.5. Colony of Culex pipiens

*Culex pipiens molestus* larvae were provided from the Medical and Molecular Entomology Section, Faculty of Science, Benha University, Egypt. Mosquito larvae were reared at 27 ± 2 °C and 75–80%, relative humidity (RH) and under a photoperiod of 14:10 h (light/dark) in the insectary room for six generations, according to [32].

### 2.6. In Vitro Larvicidal Efficacy

The crude oils and NLC Nanoformulation were tested to evaluate their larvicidal activity against early 4th larval instar *C. pipiens* [33]. About 400 mL of oil were mixed with tween 20 to arrive at tween concentrations of 0.05% (*v*/*v*). Twenty mosquito larvae were placed in a 500 mL glass beaker containing 250 mL. Concentrations of essential oil and NLC formulation (125, 250, 500, 1000, and 2000 ppm) were performed according to [34]. Each experiment was conducted three times, with the control group receiving only the solvent. Larval mortality was measured at 24 and 48 h after treatment (PT).

### 2.7. Larvicidal Field Evaluation

Fennel and green tea oils and their NLC formulation were evaluated against mosquito larvae in stagnant water ditches (average 350 m × 4.5 m × 0.65 m) and mosquito adults at different homes in Barakta village, Qalyubiya Governorate, Egypt. The larval breeding ditches were selected to contain steady water and a high mosquito stage density. Each fennel and tea oil and its NLC formulation (500 mL/m^2^) were applied to the breeding sites with dose = LC_95_ X2 (8932.68, 1659.34, 11,729.2, and 2251.24 ppm, respectively). The essential oils were dissolved with tween 20 prior to the application to ditches (about 400 mL Oil mixed with 0.2 mL of a solution of 0.05% tween 20 *v*/*v*). The results showed a low larval density in the small ditches in Barkata village post-treatment PT.

For each treatment, three replicates were performed. Before treatment, mosquito larvae were sampled from each site, and mosquito samples were taken every day for a week. To examine the efficacy of the selected larvicides on the mosquito population, fourth instar larvae were collected in field water from each site using an enamel pad (450 mL) in each larvicidal treated and transported to the laboratory for mosquito larval counting to determine the mortality, and count alive larvae until adulthood to determine the persistence of tested materials [35].

### 2.8. In Vitro Adulticidal Efficacy

Adult mosquito susceptibility testing was carried out using modified CDC bottle bioassays [36]. Pure ethanol was used as a solvent for preparing each oil in five concentrations (2, 5, 10, 15, and 20%). The NLC-loaded oils were prepared at different concentrations (0.5, 1, 2, 3, and 4%). The bottles were coated with the desired concentrations of NLC-loaded oils and left open overnight at 28 ± 2 °C to evaporate the solvent. For each concentration, three bottles were used. Adult mosquitoes (10–15 number, 3–4 days old) were picked up from the cage using a manual aspirator. The exposure times were 10, 20, 30, 40, and 60 min then adults were transferred from the bottles to separate paper cups containing a 10% sucrose solution. For each concentration, three replicates were performed. Mortality was measured 24 h after the mosquito was placed in separate paper cups.

### 2.9. Adulticidal Field Evaluation

The efficacy and stability testing of fennel and green tea oils and its NLC formulation on adult mosquitoes was carried out in multiple homes in Barkata village containing animal pens located near irrigation canals according to the guidelines of WHO [37]. The fennel and green tea oils and their NLC formulation (LC_95_ X2), 100 mL/m^2^ (0.5–1 L/room) were sprayed into three rooms in each selected home for 5 min. Three rooms were sprayed with dechlorinated water as a control group. The reduction of adult mosquitoes was calculated according to [38], before spraying the tested materials in the rooms, white cloth was spread on the floor surface of the room to collect the dead mosquitoes after spraying to know the adult density and in the untreated rooms, the adult mosquitoes were collected through the light CDC and put in the freezer (−20 °C) for 10 min to count the mosquitoes.

### 2.10. Efficacy of Selected Oils against Non-Target Predators

Fennel, green tea oils and their NLC formulation were tested against some of the prevalent predators in the mosquito breeding habitats, like *Gambusia affinis*, *Cybister tripunctatus*, and *Sphaerodema urinator*, which were captured by a bystander dipper (400 mL with a long handle) and placed in plastic bags half-filled with water collected from breeding sites. Predators fasted for 24 h prior to the bioassay and were treated with LC_50_ of fennel, green tea oils and their NLC formulation with 2 predators/1 L dechlorinated water which was placed in a 5 L bottle containing 200 larvae. Three replicates were performed for each test along with controls and the observations were made at 24 h. The captured predators were identified according to Ismaieel et al. [38] and Shoukry [39], Museum of Entomology Department, Benha University in Egypt.

### 2.11. Data Analysis

The data were analyzed by the computer program PASW Statistics 2009. The one-way analysis of variance (ANOVA), Tukey’s range test, and Probit analysis (SPSS version 22) were performed.

The percent reduction in larval and pupal density was determined according to [40] using the following formula:

% reduction = 100 − {(C1 × T2)/(C2 × T1)} × 100

where C1 is pre-treatment immature density in control habitats, C2 is post-treatment immature density in control habitats, T1 is pre-treatment immature density in treated habitats and T2 is post-treatment immature density in treated habitats.

## 3. Results

### 3.1. Synthesis of Free and Loaded NLCs

#### 3.1.1. Droplet Size and Surface Charge

**Figure 1:**
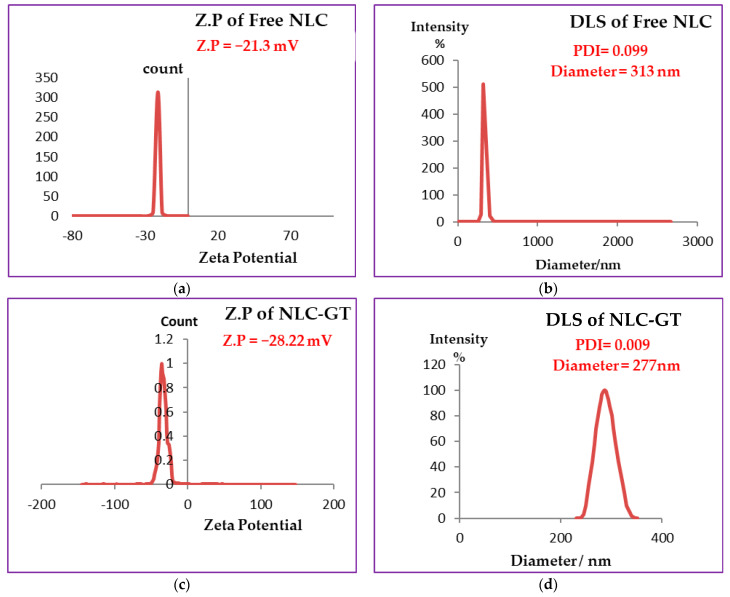

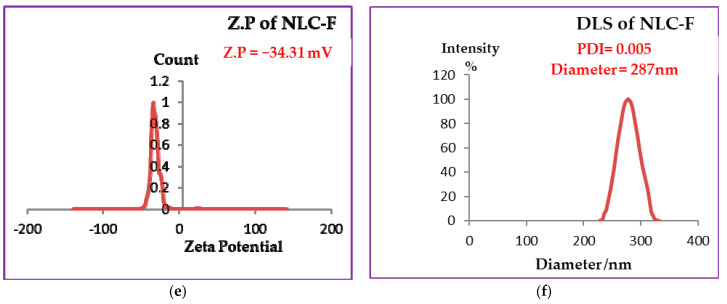
Measurements of DLS and Zeta Potential. (**a**) Represents zeta potential and (**b**) particle size of free NLC(without oils). (**c**) Represents zeta potential and (**d**) particle size of NLC-GT (loaded with green tea oil). (**e**) Represents zeta potential and (**f**) particle size of NLC–F (loaded with fennel oil).

DLS measures the z-average mean of the nanoparticles [41]. The DLS mechanism evaluates the Brownian motion of the particles. Small droplets move faster than large ones. Figure 1b,d,f showed the average particle size and PDI of free NLC, NLC-GT and NLC-F, respectively. NLC encapsulated green tea oil exhibited the smallest particle size (277 nm) and NLC encapsulated fennel oil showed a particle size of (287 nm), while free NLC (actually NLC) had the largest particle size, perhaps because free NLC was formed only by solid lipid nanoparticles (SLN) without the liquid oil phase found in NLC-GT and NLC-F. Due to the common disadvantages of NLC, such as a higher tendency to gelation, lipid particle growth [42,43] and their low incorporation rate because of the crystalline structure of the solid lipid [44,45], this may explain the reason why the particle size of NLCs is encouraged to be larger than to NLCs.

PDI represents the homogeneity or heterogeneity of the nanoparticles and is defined as the ratio mass average molecular mass to the number average molecular mass, as the ratio becomes smaller it means more homogeneity. A small value of PDI indicated narrow distribution [46]. The range of PDI values varied from 0 for monomodal or monodispersed to 1 for width size distribution when the value of PDI > 0.5 indicated a broad distribution [47]. All three types of NLCs prepared showed very low PDI values (less than 0.1) 0.099, 0.006 and 0.009 for free NLC, NLC-GT and NLC-F, respectively. The obtained results confirmed that the synthesized NLCs have homogenous narrow particle sizes with monomodal particle distribution Table 1.

**Figure 2:**
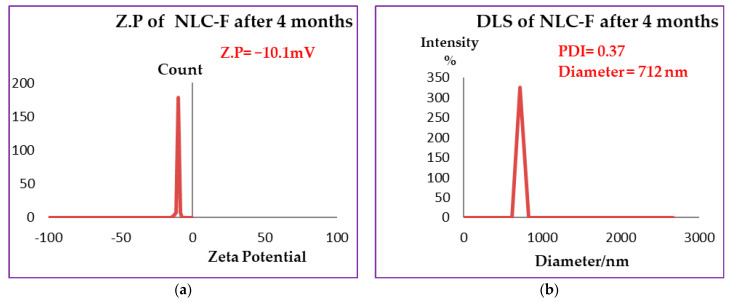
Zeta Potential (**a**) and DLS (**b**) measurements of NLC-F after four months.

NLCs surface charge was measured by zeta potential, where the best values of Z.P are >+30 and <−30. When the values were more positive than +30 and more negative than −30 stability is favored due to the repulsive forces between the same charged particles and consequently between droplets [48] that enhanced the stability by preventing or decreasing aggregation or agglomeration. According to Figure 1a,c,e the values of Z.P of free NLC = −21.3 mV, NLC-GT = −28.22 mV and NLC-F = −34.31 mV. The higher values of zeta (NLC-F = −34.31 mV) indicated more repulsion force generated between droplets [49], to prevent aggregation and provide good physical stability. Fennel encapsulated NLC showed the best stability whereas the other nanoformulations NLC-GT and Free NLC also exhibited good stability. The stability order of the prepared systems is NLC-F > NLC-GT > free NLC is shown in Table 1. The value of zeta potential decreased while DLS and PDI increased due to the aggregations occurring that made microemulsion particles become larger, affecting their mobility by making them slower, which consequently led to increasing PDI and DLS values and the system became more heterogeneous. After 4 months, the DLS and Z.P measurements of NLC-F were re-evaluated, and the results are shown in Figure 2. The DLS increased from 287 nm to 712 nm and PDI increased from 0.005 to 0.37 while Z.P decreased from −34.31 to −10.1 mV. Although, the increasing of PDI and DLS, microemulsion still has some type of stability [50].

#### 3.1.2. NLC Surface Morphology by Transmission Electron Microscope (HR-TEM)

**Figure 3:**
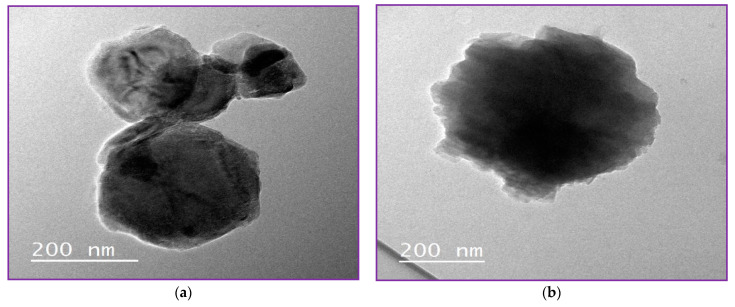

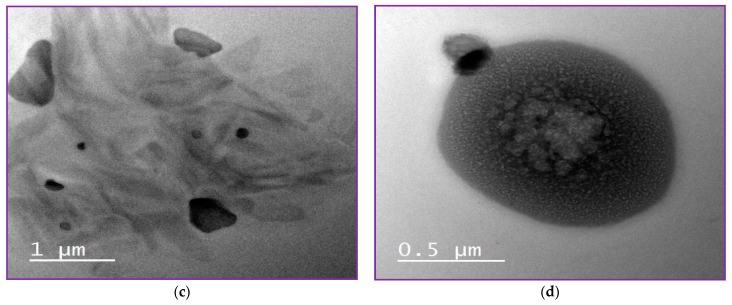
NLCs Morphology by TEM. (**a**,**b**) showed morphology of free NLC, (**c**) showed morphology of NLC-GT and (**d**) showed NLC-F.

High-resolution transmission microscope is one of the most highly effective ways to describe the structural detail, size distribution and internal morphology of NLCs or nanoparticles [3]. Free NLC, NLC-GT and NLC-F exhibited morphological characteristics similar to those previously reported [3]. Figure 3 demonstrated the morphology of the prepared NLCs and showed regular spherical shapes with particle size in the range of 200 nm, which is inconsistent with PDI and Z.P obtained from Dynamic light scattering (DLS). Figure 3d clearly showed the encapsulation of fennel and NLC with a droplet size of 500 nm, whereas the inner layer represented the oil droplet and the outer layer represented the lipid nanostructured carrier (NLC). The largeness of some droplets described by TEM as in NLC-F (0.5 µm = 500 nm) is not confused with the particle size presented by DLS. In HR-TEM, every single droplet was imaged and measured separately, while DLS was measured based on average ratio, by the mean DLS measuring the average size, not specific particle size.

### 3.2. Larvicidal Laboratory Evaluation

The larvicidal effects of oils and their nanoformulations were evaluated against the early fourth larvae of *C. pipiens*. The mortality (MO) % post-treatment (PT) with 2000 ppm for 24 h with fennel oil and NLC-F and free-NLC reached 85%, 100%, and 50.0% (Table 2), whereas MO% for green tea oil and NLC-GT were 80% and 100%, respectively (Table 3). The LC_50_ and LC_95_ values were calculated for fennel oil (643.81 and 4466.34 ppm), NLC-F (251.71 and 829.67 ppm), green tea oil (746.52 and 5864.60 ppm), and NLC-GT (278.63 and 1125.62 ppm) (Table 4). Furthermore, data showed that NLC-F was more highly effective than fennel oil and free-NLC, whereas the mean of treatment % was 41.39%, 24.26%, and 16.61%, respectively. Similarly, data showed that NLC-GT was more highly effective than green tea oil and free-NLC, whereas the mean of treatment percent was 39.39%, 22.26%, and 16.61%, respectively.

### 3.3. Larvicidal Field Evaluation

The field evaluation of larvicides was carried out using LC_95_ X2 doses of fennel oil and NLC-F (8932.68 and 1659.34 ppm, respectively) and for green tea oil and NLC-GT (11,729.2 and 2251.24 ppm, respectively), and only dechlorinated water was used at the control location, where the results showed a low larval density in the small ditches in Barkata village PT.

**Figure 4:**
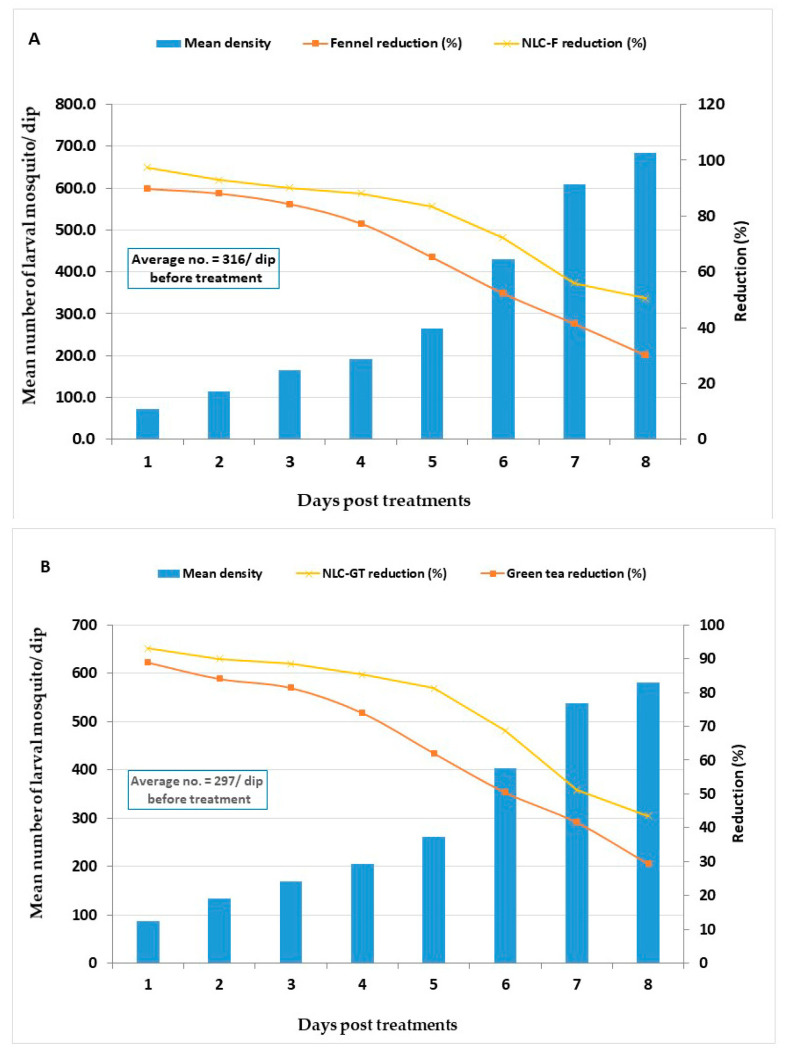
Field evaluation for larvicidal efficacy of fennel oil and NLC-F (**A**) Nanoformulation, green tea oil, and NLC-GT (**B**) Nanoformulation treated at a dose of LC_95_ X2 (8932.68 and 1659.34 ppm), and (11,729.2 and 2251.24 ppm), respectively, in larval breeding sites.

Treatments reduced larval density and the larval reduction percent of fennel oil and NLC-F reaching 89.8 and 97.4%, 24 h PT with persistence to eight days PT (Figure 4). The corresponding values for green tea oil and NLC-GT reached 89% and 93%, with persistence to seven days PT, respectively (Figure 4).

### 3.4. In Vitro Adulticidal Effect

The adulticidal effects of the applied materials were evaluated against *C. pipiens* adults (3–4 old days), and the results showed that NLC-F (100% MO) was more effective than fennel oil (90.0%) and Free-NLC, 24 h PT, respectively, and their LC_95_ values were 2.18%, 12.74% and 15.67% **(**Table 5). Furthermore, data showed that NLC-GT (100% MO) was more effective than green tea oil (83.33%), 24 h PT, respectively, and their LC_95_ values were 2.68% and 19.18%, respectively (Table 6).

### 3.5. Adult Field Mosquito Experiments

**Figure 5:**
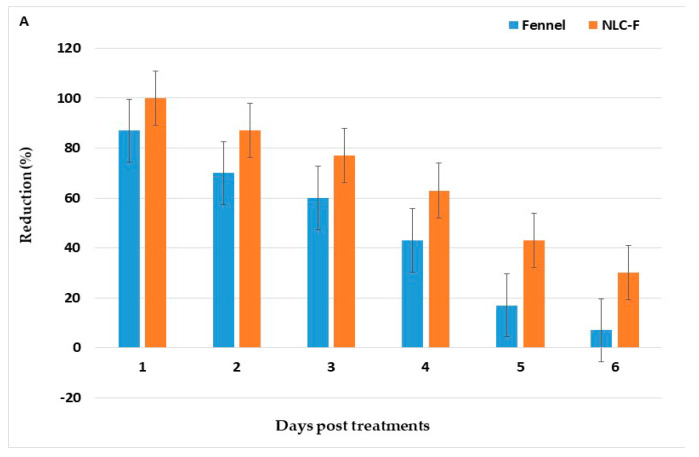

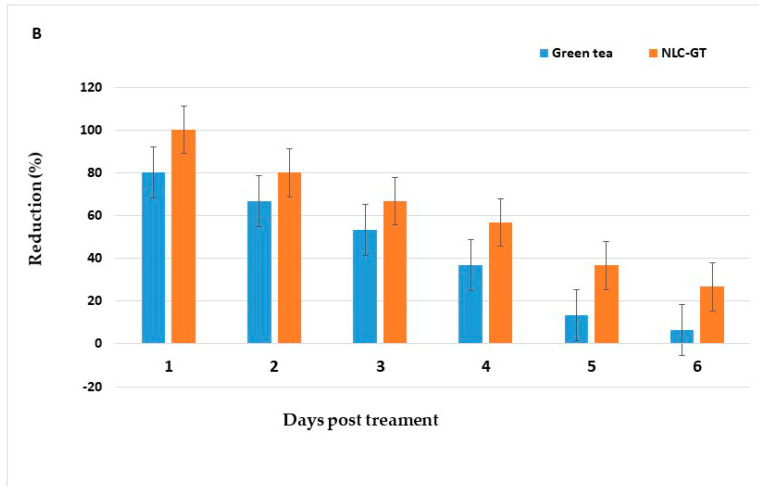
Persistence of Fennel oil and its NLN-F (**A**), and green tea oil and its NLN-GT (**B**) against adult mosquitoes in treated homes, 30 min post-exposure for 6 days.

After spraying with LC_95_ X2 for 15 min [37], the reduction of adult density PT with fennel oil, NLC-F, and green tea oil, NLC-GT were 83.6%, 100%, 79.1%, and 100%, respectively, with persistence (>50%) lasted for three days (Table 7, Figure 5).

### 3.6. The Efficacy of Oils and NLC against Non-Target Predators

The efficacy of the oils and their NLC formulation against some predators as non-target insects were investigated, including *C. tripunctatus, G. affinis*, and *S. urinator* after being treated with LC_50_ of fennel (643.81), green tea oils (746.52) and NLC formulation (NLC-F = 251.71 and NLC-GT = 278.63), respectively. Data showed no significant difference between the mean predation for both fennel oil and NLC-F and all selected predators. While in the case of green tea oil and NLC-GT, the predation rate of the beetle, *C. tripunctatus* increased (66% and 68.3%, respectively (Table 8). Mosquitofish, *G. affinis*, and *S. urinator* bugs were not affected in both oil and NLC for both oils.

## 4. Discussion

Plant-derived insecticides effectively controlled the larvae and pupae of mosquitoes, acting as a good alternative to synthetic pesticides [51,52]. The current study confirmed that the *F. vulgare* (fennel oil) and *C. sinensis* (green tea) oils have larvicidal and adulticidal activity and their NLC nanoformulations increased their efficacy and persistence.

The novel fennel NLC nanocomposites were more effective in controlling the larvae and adults of *C. pipiens* (LC_50_ = 251.71 ppm and 0.25%, respectively) than crude fennel oil (LC_50_ = 643.81 ppm and 2.80%, respectively). While NLC-F had a light effect on larvae and adults according to the mean treatments and ensures that the aquatic environment is not polluted with the nanocomposite’s additives.

Our data revealed that fennel oil is more effective than green tea oil in controlling the larvae and adults of *C. pipiens* mosquitoes. Furthermore, when fennel oil was converted into a nanoemulsion, its efficiency increased by more than 10 times the crude oil.

Similar to our findings, several researchers have demonstrated the apparent efficacy of fennel oil against the larvae and adults of *Culex quinquefasciatus* [53,54], *Anopheles atroparvus* [55], and *Aedes aegypti* [56]. In another study, fennel oil (40 mg/L), was sufficient to cause 50% death in the second larval stage of *C. pipiens* after two h PT [57]. Moreover, a concentration of 60 mg/L resulted in 90% death of fourth instar larvae after 4 h PT indicating the efficiency of the oil within a short period.

The novel green tea NLC nanocomposites were more effective controlled larvae and adults of *C. pipiens* (LC_50_ = 278.63 ppm and 0.40%, respectively) than crude oil green tea oil (LC_50_ = 746.52 ppm and 4.21%, respectively).

Our data agree with a similar study that found that *C. sinensis* markedly malformed larvae and pupae (LC_50_ = 540 and 600 ppm, respectively), and provided protection (100%) from the bites of hungry females at a dose of 6 mg/cm^2^. Moreover, green tea leaf extract showed practical larvicide effects against *Anopheles arabiensis* and *Anopheles gambiae* [58], causing 100% larval mortality at 1000 ppm, and adult repellent effects against *C. Pipiens*. Green tea is also an effective larvicide against *Drosophila prosaltans* due to caffeine and other organic compounds [59].

Local essential oils such as *Nigella sativa*, *Allium cepa*, and *Sesamum indicum*, had a larvicidal effect against the laboratory and field *C. pipiens* strains in Egypt (LC_50_ = 247.99 and 108.63; 32.11 and 2.87; and 673.22 and 143.87 ppm, respectively); such oils adversely affected pupae and adult emergence rates and induced abnormalities in larval development [29]. In addition, the oils of fenugreek (*Trigonella foenum-grecum*), mustard (*Brassica compestris*), ground almond (*Cyperus esculentus*), frankincense (*Boswellia serrata*), watercress (*Eruca sativa*), and parsley (*Carum Petroselinum*) effectively reduced mosquito larvae *C. pipiens* larvae (LC_50_ = 32.42, 71.37, 47.17, 83.36, 86.06, and 152.94 ppm) [60].

The current work indicated that fennel and green tea oils and their nanoformulations reduced larval and adult mosquito density in the field. A similar, adulticidal effect was observed when EOs were applied topically to laboratory and natural field strains of *Aedes aegypti*, and the laboratory strain was more susceptible than the natural field strain [61].

It was reviewed that most of the plant-fabricated polydisperse metal nanoparticles were highly effective as mosquito larvicides, insecticides, and acaricides at very low LC_50_ values (1–30 mg/L) [62]. *Nicandra physalodes* Ag NPs used against *Anopheles stephensi,* *A. aegypti* and *C. quinquefasciatus* [24]; *Zorina diphylla* AgNP against *Anopheles subpictus, Aedes albopictus*, and *Culex tritaeniorhynchus* [63]; *Cyprus rotundas NgNPs* against *A. albopictus*, *An. stephensi* and *C. quinquefasciatus* [64].

Some oils act as adult mosquito repellents; *Zanthoxylum piperitum* oil alone and oil plus 5% vanillin repelled laboratory-reared female *A. aegypti* (median protection times = 1.5 and 2.5 h, respectively). Under field conditions, *Z. piperitum* oil + 5% vanillin was found to provide better protection against *Aedes gardnerii*, *Anopheles barbirostris*, *Armigeres subalbatus*, *C. tritaeniorhynchus*, *C. gelidus*, *C. Vishnu* group, and *Mansonia uniformis*) than 25% DEET + 5% vanillin [65].

Reduced adult emergence rates of *Aedes aegypti* and *C. quinquefasciatus* were recorded PT with aqueous leaf extract of *Adiantum raddianum* and its green synthesized AgNPs [66]. Silver, protein-lipid, nanoparticles (Ag-PL NPS) (core-shell) fabricated from the seed extract from an almond tree, *Sterculia foetida* (LC_50_ < 4.5 ppm) against larvae of *A. aegypti*, *Anopheles stephensi*, and *C. quinquefasciatus* [67].

Our results revealed that the larval reduction percent of NLC-F was more efficacy in reduced larval density (97.4%) than fennel oil (89.8%) and their effect lasted (reduction% > 50%) for 8 and 7 days PT, respectively. In comparison, those of green tea oil and NLC-GT were 89.0% and 93%, respectively, 24 h PT and persisted for 6 and 7 days PT, respectively.

On the other hand, the reduction of adult density PT with fennel oil, NLC-F, green tea oil, and NLC-GT reached 83.6%, 100%, 79.1%, and 100%, respectively, and was effective for three days. According to our knowledge, there was no previous filed application of nanoparticles against mosquitoes.

Ecofriendly EOs are highly effective in killing mosquito larvae and insects in general. Plant compounds such as flavonoids, alkaloids, esters, glycosides, and fatty acids have anti-insect effects on the chemical compounds used in the elimination of insects in various ways [30,68], such as repellents [69], attractants [70], feeding deterrents/antifeedants, toxicants, growth retardants, and chemosterilants [25,29].

Because of its psycho-activity and health benefits, tea leaves, *C. Sinensis* are used to produce a non-alcoholic drink worldwide [58]. The immature tea leaves are high in methylxanthines (caffeine, theophylline), catechins (catechin, gallocatechin, catechin gallate), flavonoids, vitamins, proteins, glycosides (kaempferol, myricetin) [71]. Catechins have antiviral, antibacterial, antimalarial, anticarcinogenic, antioxidant, anti-inflammatory, anti-aging, and anti-arthritis [72,73].

In a previous recent study, Baz et al. [74] revealed that major compounds of *F. vulgare* oil (GC/MS) were Estragole (70.36%); Limonene (8.96%), where the major components of *C. sinensis* oil were Gallic acid (1674 µg/mL), Catechin (421 µg/mL), Coffeic acid (678 µg/mL), Coumaric acid (566 µg/mL), and Naringenin (178 µg/mL). It had been noted that proanthocyanidins were the most abundant bioactive chemical in *C. Sinensis* leaf extract. Fennel oil contains estragole (70.36%) and limonene (8.96%) (unpublished work) [58]. Estradiol is toxic to adult fruit flies, *Ceratitis capitata* [75], and limonene is a cyclic monoterpene with insecticidal effect [76].

Several researchers confirmed that the main components of Egyptian fennel essential oil include estragol, l-fenchone, limonene, and trans-anethole through GC-MS analysis [77,78]. The chemical composition of the tested fennel essential oil matched European Pharmacopoeia criteria for tA, α-pinene, limonene and estragole [79]. Larvicidal activity of the fennel EOs and its major constituents ((-)-limonene) against third instar larvae of *A. aegypti* for 24 h were evaluated, whereas mortality reached 99% at 37.1 and 52.4 µL L-l of fennel EOs from Cape Verde and Portugal areas, respectively [56].

Some studies reveal the mode of action of the synthesized green nanoparticles, which may be related to the penetration of insect exoskeleton and the ability of the nanoparticles to bind to the sulfur elemental with proteins or to DNA phosphorylation to rapid denaturation of saturation. It has been recently recorded that nanoparticles enter the membrane of mosquito larvae and then into their gut and damage their DNA-binding pattern [64]. Benelli, Caselli and Canale [62] reviewed the mechanisms of action of nanoparticles against insects. Silver and graphene oxide nanoparticles affected insect antioxidant and detoxifying enzymes inducing oxidative stress and cell death.

Due to their excellent predation effectiveness, mosquito predators such as *Gambusia* sp. are released worldwide for biological mosquito control [62]. This work indicated the safety of the applied materials against three non-target organisms. Furthermore, several studies about the acute toxicity of nanoparticles against the aquatic non-target species did not detect toxicity of silver nanoparticles produced using plant extracts toxic to mosquito larvae [80].

This study indicated that the predation rates of *C. tripunctatus* were slightly increased after subjecting to NLC-GT and crude oils, respectively, when compared to NLC-F and oils and control group. In contrast, a noticed increase in the predation rate of the tadpoles, *Hoplobatrachus tigerinus*, against larvae of *A. aegypti*, in the laboratory and in an aquatic environment, when treated with ultra-low doses of AgNP [81]. AgNP of *Nicandra physalodes* was safe for the non-target aquatic organism *Diplonychus indicus (*LC_50_ and LC_90_ values were 1032.81 and 19,076.59 μg/mL, respectively) [24]. *Zorina diphylla* AgNP is safe for some non-target organisms as *Chironomus circumdatus*, *Anisops bouvieri* and *Gambusia affinis* (LC_50_ = 613.11–6903.93 μg/mL) [63].

## 5. Conclusions

NLCs formulation blend with essential oils was prepared successfully with unique properties of size, morphology, and stability. Mosquitoes are essential vectors of serious diseases representing global concerns. For the first time against *C. pipiens,* our findings revealed the in vitro field larvicidal, adulticidal effects of fennel and green tea oils and their NLC microsphere. Fennel and green tea nanoemulsion reduced the dose needed to kill mosquitoes and increased their persistence in the field condition. Therefore, they could be used for integrated mosquito control programs. After revealing their ecotoxicological profile, larger-scale studies are needed to develop eco-friendly insecticides for enhanced efficacy and minimal dose.

## Figures and Tables

**Table 1:** Dynamic light scattering size distribution, zeta potential, and polydispersity index of free NLC, NLC-GT and NLC-F.

System	Size (DLS)	Zeta Potential	PDI
Free-NLC	313 nm	−21.30 mv	0.099
NLC-GT	277 nm	−28.22 mv	0.009
NLC-F	287 nm	−34.31 mv	0.005

**Table 2:** Larvicidal effects of fennel oil and its NLC against *Culex pipiens,* 24 h post-treatment.

Oil Name	Tested Materials	Time (h)	Mortality % (Mean ± SE) * of Mosquito Larvae					
			0 **	125	250	500	1000	2000
Fennel (*Foeniculum vulgare*)	Oil	0.5	0.00 ± 0.00 ^aE^	0.00 ± 0.00 ^eE^	1.67 ± 1.67 ^eD^	3.33 ± 1.67 ^eC^	8.33 ± 1.67 ^eB^	20.00 ± 0.00 ^eA^
		2	0.00 ± 0.00 ^aF^	1.67 ± 1.67 ^dE^	3.33 ± 1.67 ^dD^	8.33 ± 1.67 ^dC^	21.67 ± 1.67 ^dB^	33.33 ± 3.33 ^dA^
		8	0.00 ± 0.00 ^aF^	3.33 ± 1.67 ^cE^	8.33 ± 1.67 ^cD^	18.33 ± 1.67 ^cC^	41.67 ± 1.67 ^cB^	60.00 ± 2.89 ^cA^
		24	0.00 ± 0.00 ^aF^	8.33 ± 1.67 ^bE^	21.67 ± 1.67 ^bD^	41.67 ± 3.33 ^bC^	61.67 ± 4.41 ^bB^	85.00 ± 2.89 ^bA^
		48	0.00 ± 0.00 ^aF^	13.33 ± 1.67 ^aE^	30.00 ± 2.89 ^aD^	58.33 ± 1.67 ^aC^	81.67 ± 1.67 ^aB^	100.00 ± 0.00 ^aA^
	NLC-F	0.5	0.00 ± 0.00 ^aF^	6.67 ± 1.67 ^eE^	11.67 ± 1.67 ^eD^	18.33 ± 1.67 ^eC^	33.33 ± 1.67 ^dB^	40.00 ± 2.89 ^dA^
		2	0.00 ± 0.00 ^aF^	8.33 ± 1.67 ^dE^	15.00 ± 0.00 ^dD^	25.00 ± 2.89 ^dC^	43.33 ± 4.41 ^cB^	58.33 ± 1.67 ^cA^
		8	0.00 ± 0.00 ^aF^	13.33 ± 1.67 ^cE^	33.33 ± 1.67 ^cD^	56.67 ± 1.67 ^cC^	78.33 ± 1.67 ^bB^	90.00 ± 2.89 ^bA^
		24	0.00 ± 0.00 ^aE^	20.00 ± 2.89 ^bD^	46.67 ± 3.33 ^bC^	78.33 ± 1.67 ^bB^	100.00 ± 0.00 ^aA^	100.00 ± 0.00 ^aA^
		48	0.00 ± 0.00 ^aE^	25.00 ± 2.89 ^aD^	56.67 ± 4.41 ^aC^	83.33 ± 3.33 ^aB^	100.00 ± 0.00 ^aA^	100.00 ± 0.00 ^aA^
	Free-NLC	0.5	0.00 ± 0.00 ^aE^	0.00 ± 0.00 ^eE^	3.33 ± 1.67 ^eD^	5.0 ± 0.00 ^eC^	8.33 ± 1.67 ^eB^	16.67 ± 1.67 ^eA^
		2	0.00 ± 0.00 ^aF^	1.67 ± 1.67 ^dE^	6.67 ± 1.67 ^dD^	8.33 ± 1.67 ^dC^	16.67 ± 1.67 ^dB^	26.67 ± 1.67 ^dA^
		8	0.00 ± 0.00 ^aF^	5.00 ± 0.00 ^cE^	11.67 ± 1.67 ^cD^	18.33 ± 4.41 ^cC^	26.67 ± 3.33 ^cB^	38.33 ± 3.33 ^cA^
		24	0.00 ± 0.00 ^aF^	8.33 ± 1.67 ^bE^	15.00 ± 2.89 ^bD^	25.0 ± 5.77 ^bC^	40.0 ± 2.89 ^bBB^	50.00 ± 2.89 ^bA^
		48	0.00 ± 0.00 ^aF^	11.67 ± 1.67 ^aE^	20.00 ± 2.89 ^aD^	31.67 ± 1.67 ^aC^	48.33 ± 6.01 ^aB^	58.33 ± 1.67 ^aA^

* Different capital letters (row) and lowercase letters (column) indicate that the differences between the groups are significant by (one-way ANOVA, Tukey’s range test, *p* > 0.05). ** LC values = ppm.

**Table 3:** Larvicidal effects of green tea oil and its NLC against *Culex pipiens,* 24 h post-treatment.

Oil Name	Tested Materials	Time (h)	Mortality % (Mean ± SE) * of Mosquito Larvae					
			0 **	125	250	500	1000	2000
Green tea (*Camellia sinensis*)	Oil	0.5	0.00 ± 0.00 ^eD^	0.00 ± 0.00 ^eD^	0.00 ± 0.00 ^eD^	3.33 ± 1.67 ^eC^	8.33 ± 1.67 ^eB^	13.33 ± 1.67 ^eA^
		2	0.00 ± 0.00 ^aF^	1.67 ± 1.67 ^dE^	3.33 ± 1.67 ^dD^	6.67 ± 1.67 ^dC^	18.33 ± 1.67 ^dB^	28.33 ± 1.67 ^dA^
		8	0.00 ± 0.00 ^aF^	3.33 ± 1.67 ^cE^	8.33 ± 1.67 ^cD^	16.67 ± 1.67 ^cC^	35.00 ± 2.89 ^cB^	53.33 ± 1.67 ^cA^
		24	0.00 ± 0.00 ^aF^	8.33 ± 1.67 ^bE^	18.33 ± 1.67 ^bD^	38.33 ± 1.67 ^bC^	56.67 ± 3.33 ^bB^	80.00 ± 2.89 ^bA^
		48	0.00 ± 0.00 ^aF^	11.67 ± 1.67 ^aE^	26.67 ± 1.67 ^aD^	55.00 ± 2.89 ^aC^	80.00 ± 2.89 ^aB^	100.00 ± 0.00 ^aA^
	NLC-GT	0.5	0.00 ± 0.00 ^aF^	5.00 ± 0.00 ^eE^	10.00 ± 0.00 ^eD^	16.67 ± 1.67 ^eC^	28.33 ± 1.67 ^eB^	35.00 ± 2.89 ^dA^
		2	0.00 ± 0.00 ^aF^	8.33 ± 1.67 ^dE^	13.33 ± 1.67 ^dD^	21.67 ± 1.67 ^dC^	40.00 ± 2.89 ^dB^	56.67 ± 1.67 ^cA^
		8	0.00 ± 0.00 ^aF^	11.67 ± 1.67 ^cE^	33.33 ± 1.67 ^cD^	50.00 ± 2.89 ^cC^	75.00 ± 2.89 ^cB^	88.33 ± 1.67 ^bA^
		24	0.00 ± 0.00 ^aF^	20.00 ± 2.89 ^bE^	45.00 ± 2.89 ^bD^	70.00 ± 2.89 ^bC^	95.00 ± 2.89 ^bB^	100.00 ± 0.00 ^aA^
		48	0.00 ± 0.00 ^aF^	23.33 ± 1.67 ^aDE^	55.00 ± 2.89 ^aC^	80.00 ± 2.89 ^aB^	100.00 ± 0.00 ^aA^	100.00 ± 0.00 ^aA^
	Free-NLC	0.5	0.00 ± 0.00 ^aE^	0.00 ± 0.00 ^eE^	3.33 ± 1.67 ^eD^	5.0 ± 0.00 ^eC^	8.33 ± 1.67 ^eB^	16.67 ± 1.67 ^eA^
		2	0.00 ± 0.00 ^aF^	1.67 ± 1.67 ^dE^	6.67 ± 1.67 ^dD^	8.33 ± 1.67 ^dC^	16.67 ± 1.67 ^dB^	26.67 ± 1.67 ^dA^
		8	0.00 ± 0.00 ^aF^	5.00 ± 0.00 ^cE^	11.67 ± 1.67 ^cD^	18.33 ± 4.41 ^cC^	26.67 ± 3.33 ^cB^	38.33 ± 3.33 ^cA^
		24	0.00 ± 0.00 ^aF^	8.33 ± 1.67 ^bE^	15.00 ± 2.89 ^bD^	25.0 ± 5.77 ^bC^	40.0 ± 2.89 ^bBB^	50.00 ± 2.89 ^bA^
		48	0.00 ± 0.00 ^aF^	11.67 ± 1.67 ^aE^	20.00 ± 2.89 ^aD^	31.67 ± 1.67 ^aC^	48.33 ± 6.01 ^aB^	58.33 ± 1.67 ^aA^

* Different capital letters (row) and lowercase letters (column) indicate that the differences between the groups are significant by (one-way ANOVA, Tukey’s range test, *p* > 0.05). ** LC values = ppm.

**Table 4:** LC_50_ and LC_90_ (ppm) values of fennel, green tea oils and its NLC formulations against *Culex pipiens* larvae, 24 h post-treatment.

Oil Name	Tested Materials	LC_50_ (95%CI) *	LC_90_ (95%CI)	LC_95_ (95%CI)	Chi (Sig)	Equation	R2
Fennel (*Foeniculum vulgare*)	Oil	643.81 (556.16–751.24)	2911.80 (2220.73–4188.70	4466.34 (3228.20–6943.48)	3.102 (0.089 a)	Y = −1.955 + 0.16 E – 3 * X	0.941
	NLC-F	251.71 (224.06–280.58)	637.52 (549.36–771.17)	829.67 (695.16–1046.64)	9.368 (0.014 a)	Y = −3.175 + 0.26 E – 3 * X	0.922
Green tea (*Camellia sinensis*)	Oil	746.52 (638.96–885.48)	3719.81 (2731.38–5681.86)	5864.60 (4052.26–9792.05)	2.761 (0.091 a)	Y = −1.837 + 0.162 E – 3 * X	0.959
	NLC-GT	278.63 (243.05–311.57)	825.55 (700.73–1016.21)	1125.62 (925.22–1452.73)	7.261 (0.035 a)	Y = −2.698 + 0.212 E – 3 * X	0.919

* LC values = ppm.

**Table 5:** The adulticidal effects of Fennel oil and its NLC formulations against adult *Culex pipiens*, 24 h post-treatment.

Oil Name	Tested Materials	Conc. (%)	Mortality% (Mean ± SE)	LC_50_ (95%CI) *	LC_90_ (95%CI)	LC_95_ (95%CI)	Chi (Sig)	Equation	R^2^
Fennel (*Foeniculum vulgare*)	Oil	0	3.33 ± 3.33 ^e^	2.805 (0.285–3.685)	6.236 (4.173–12.699)	12.748 (7.332–35.19)	7.656 (0.053 ^a^)	Y = 1.1699 + 0.185 * x	0.991
		2.0	36.67 ± 6.67 ^d^						
		5.0	56.67 ± 3.33 ^c^						
		10.0	70.00 ± 5.77 ^b^						
		15.0	83.33 ± 3.33 ^a^						
		20.0	90.00 ± 5.77 ^a^						
	NLC-F	0.0	6.67 ± 3.33 ^c^	0.259 (0.131–0.377)	1.599 (1.1019–1.8134)	2.180 (1.667–3.414)	5.387 (0.067 ^a^)	Y = −1.780 + 0.291 * X	0.927
		0.5	73.33 ± 3.33 ^b^						
		1.0	80.00 ± 0.00 ^b^						
		2.0	93.33 ± 3.33 ^a^						
		3.0	100.00 ± 0.00 ^a^						
		4.0	100.00 ± 0.00 ^a^						
	Free-NLC	0.0	0.00 ± 0.00 ^f^	9.630 (4.386–12.562)	23.171 (10.232–64.924)	44.196 (21.346–99.819)	5.005 (0.017 ^a^)	Y = 1.1899 + 0.221 * x	0.973
		2.0	20.00 ± 0.00 ^e^						
		5.0	33.33 ± 6.67 ^d^						
		10.0	40.00 ± 5.77 ^c^						
		15.0	53.33 ± 3.33 ^b^						
		20.0	66.67 ± 8.82 ^a^						

The different lowercase letters indicate that the differences between the groups are significant by (one-way ANOVA, Tukey’s range test, *p* < 0.05)., * LC values = %.

**Table 6:** The adulticidal effects of green oil and its NLC formulations against adult *Culex pipiens*, 24 h post-treatment.

Oil Name	Tested Materials	Time (Mint)	Mortality% (Mean ± SE)	LC_50_ (95%CI) *	LC_90_ (95%CI)	LC_95_ (95%CI)	Chi (Sig)	Equation	R^2^
Green tea (*Camellia sinensis*)	Oil	0.0	3.33 ± 3.33 ^f^	4.212 (0.599–5.425)	9.648 (6.124–21.279)	19.184 (10.555–55.446)	4.967 (0.174 ^a^)	Y = 1.216 + 0.180 * x	0.953
		2.0	23.33 ± 3.33 ^e^						
		5.0	50.00 ± 5.77 ^d^						
		10.0	60.00 ± 0.00 ^c^						
		15.0	76.67 ± 8.82 ^b^						
		20.0	83.33 ± 3.33 ^a^						
	NLC-GT	0.0	6.67 ± 3.33 ^f^	0.402 (0.322–0.562)	1.813 (1.523–2.272)	2.687 (2.160–3.664)	5.405 (0.144 ^a^)	Y = −2.126 + 0.240 * X	0.922
		0.5	66.67 ± 3.33 ^e^						
		1.0	76.67 ± 3.33 ^d^						
		2.0	86.67 ± 3.33 ^c^						
		3.0	96.67 ± 3.33 ^b^						
		4.0	100.00 ± 0.00 ^a^						
	Free-NLC	0.0	0.00 ± 0.00 ^f^	9.630 (4.385–12.562)	23.171 (10.232–64.924)	44.196 (21.346–99.819)	5.005 (0.017 ^a^)	Y = 1.1899 + 0.221 * x	0.973
		2.0	20.00 ± 0.00 ^e^						
		5.0	33.33 ± 6.67 ^d^						
		10.0	40.00 ± 5.77 ^c^						
		15.0	53.33 ± 3.33 ^b^						
		20.0	66.67 ± 8.82 ^a^						

The different lowercase letters indicate that the differences between the groups are significant by (one-way ANOVA, Tukey’s range test, *p* < 0.05)., * LC values = ppm.

**Table 7:** Field evaluation of the applied materials against adult mosquitoes in different homes, 24 h post-treatment.

Oil Name	Tested Materials	Sites	Mean Number/Site ± SE	% Mean Reduction
*Fennel* (*Foeniculum vulgare*)	Control	home 1	23.4 ± 5.41 ^a^	0.0
		home 2		
		home 3		
	Oil	home 1	4.56 ± 1.28 ^b^	83.6
		home 2		
		home 3		
	NLC-F	home 1	0.22 ± 0.22 ^b^	100.0
		home 2		
		home 3		
*Green tea* (*Camellia sinensis*)	Control	home 1	22.81 ± 5.41 ^a^	0.0
		home 2		
		home 3		
	Oil	home 1	5.89 ± 1.28 ^b^	79.1
		home 2		
		home 3		
	NLC-GT	home 1	0.22 ± 0.22 ^b^	100.0
		home 2		
		home 3		

Numbers of the same column followed by the same small letter are not significantly different (one-way ANOVA, Tukey’s range test, *p* > 0.05).

**Table 8:** The mean number of mosquito larvae, *Culex pipiens* consumed by some predators before and after treated with fennel and green tea oil, and their NLC under laboratory conditions.

Oil Name	Treatment	Mosquito Predator Types		
		*G. affinis*	*C. tripunctatus*	*S. urinator*
*Foeniculum vulgare*	Control *	76.00 ± 2.08 ^bcA^	60.67 ± 2.40 ^cB^	25.00 ± 0.58 ^aC^
	Oil	76.67 ± 2.40 ^abcA^	59.00 ± 3.51 ^cB^	23.67 ± 0.88 ^abC^
	NLC-F	76.33 ± 0.88 ^abcA^	59.67 ± 0.33 ^cB^	24.67 ± 1.33 ^abC^
*Camellia sinensis*	Oil	75.67 ± 1.76 ^cA^	66.00 ± 10.00 ^bB^	23.33 ± 2.03 ^abC^
	NLC-GT	77.67 ± 1.86 ^aA^	68.00 ± 3.06 ^aB^	24.67 ± 0.88 ^abC^

a, b and c: There is no significant difference (*p* > 0.05) between any two means, within the same column that have the same superscript letter. A, B and C: There is no significant difference (*p* > 0.05) between any two means for the same attribute, within the same row have the same superscript letter. * control without treatment. NLC-F: fennel NLC formulation; NLC-GT: green tea NLC formulation.

## Data Availability

All data analyzed during this study are included in this published article.
